# Definitions of white matter hyperintensity change: impact on estimates of progression and regression

**DOI:** 10.1136/svn-2024-003300

**Published:** 2024-10-02

**Authors:** Angela C C Jochems, Susana Muñoz Maniega, Una Clancy, Carmen Arteaga Reyes, Daniela Jaime Garcia, Maria del C. Valdés Hernández, Francesca M Chappell, Gayle Barclay, Charlotte Jardine, Donna McIntyre, Iona Gerrish, Stewart Wiseman, Michael S Stringer, Michael J Thrippleton, Fergus Doubal, Joanna M Wardlaw

**Affiliations:** 1 UK Dementia Research Institute at the University of Edinburgh, Edinburgh, UK; 2 Centre for Clinical Brain Sciences, The University of Edinburgh, Edinburgh, UK; 3 The University of Edinburgh, Edinburgh Imaging Facility, Edinburgh, UK

**Keywords:** Magnetic Resonance Imaging, Brain, Cerebrovascular Disorders, Ischemic Stroke, Lesion

## Abstract

**Background:**

White matter hyperintensity (WMH) progression is well documented; WMH regression is more contentious, which might reflect differences in defining WMH change. We compared four existing WMH change definitions in one population to determine the effect of definition on WMH regression.

**Methods:**

We recruited patients with minor non-disabling ischaemic stroke who underwent MRI 1–3 months after stroke and 1 year later. We assessed WMH volume (in absolute mL and % intracranial volume) and applied four different definitions, including two thresholds (based on SD or mL), percentile and quintile approaches.

**Results:**

In 198 participants, mean age 65.5 (SD=11.13), baseline WMH volume was 15.46 mL (SD=19.2), the mean net WMH volume change was 0.98 mL (SD=2.84), range −7.98 to +12.84 mL. Proportion regressing/stable/progressing WMH were threshold 1 (SD), 29.8%/55.6%/14.6%; threshold 2(mL), 29.8%/16.7%/53.5%; percentile approach, 28.3%/21.2%/50.5%. The quintile approach includes five groups with quintile 3 reflecting no change (N=40), quintiles 1 and 2 any WMH decrease (N=80) and quintiles 4 and 5 any WMH increase (N=78).

**Conclusions:**

Different WMH change definitions cause big differences in how participants are categorised; additionally, non-normal WMH distribution precludes use of some definitions. Consistent use of an appropriate definition would facilitate data comparisons, particularly in clinical trials of potential WMH treatments.

## Introduction

 Progression of white matter hyperintensities (WMH) of presumed vascular origin is associated with worse clinical outcomes and an increased risk of stroke and dementia.^1^ WMH regression was reported in approximately 30% of participants in a recent meta-analysis.^2^ So far, six studies have examined outcomes related to WMH regression,^3^,^8^ suggesting better clinical outcomes,^4 7^ or at least, comparable outcomes to those of people with stable WMH and better than those with progressing WMH.^6^ However, drawing robust conclusions proves challenging due to the diversity in definitions of regression, or WMH changes in general. Currently, four different approaches exist to define change, but their influence on identifying regressors/stable/progressors, or on clinical outcomes, is unclear. We compared these approaches in one population to determine differences in WMH volume changes and participant distribution between groups.

## Methods

### Participants

We recruited 230 patients with a non-disabling ischaemic stroke into a longitudinal observational study,^9^ defining non-disabling as National Institutes of Health Stroke Scale^10^ <8 and independency as a modified Ranking Score^11^ ≤2. Recruitment exclusion criteria were MRI contraindications, severe neurological, cardiac or respiratory diseases, which would preclude lying flat.^9^ Participants underwent MRI within 3 months after stroke and 1 year later. All participants provided written informed consent. Data are available on reasonable request to the corresponding author.

### Existing approaches

Table 1 summarises the WMH change approaches. A 0.25 mL change threshold was applied to define WMH regression only (threshold approach 1),^12^ or both WMH regression and progression (threshold approach 2).^3^ This threshold was reported to be the smallest visible WMH volume change, which two raters could reliably measure in 20 1.5T MRI scans.^12^ For threshold approach 1, an increase of >1 SD of the mean WMH volume change was used to define WMH progression. The third (percentile) approach divided WMH volume change into percentiles and identified the percentile with least or no WMH volume change.^4^ Percentile use was justified by the leptokurtic (non-normal) data distribution of WMH volume change, which precluded applying a 1 SD threshold.^4^ Finally, the fourth approach used quintiles of WMH change to display any net WMH volume change, from most decrease to most increase.^7^

**Table 1:** Overview of the four existing approaches to define WMH change

Approach	Regression	Stable	Progression
Threshold 1^12^	Decrease>0.25 mL	Volumes from −0.25 mL up to 1 SD (of mean WMH volume change)	Increase >1 SD (of mean WMH volume change)
Threshold 2^3^	Decrease>0.25 mL	Volumes from −0.25 mL up to +0.25 mL	Increase >0.25 mL
Percentile^4^	Percentiles below the −10th percentile of no change	Percentile of no volume change ±10 percentiles	Percentiles above the +10^th^ percentile of no change
Quintile^7^	Data are divided in quintiles reflecting any net volume change. Quintile 1 (Q1): most volume decrease; quintile 5 (Q5): most volume increase		

WMH, white matter hyperintensity.

### Imaging acquisition and analysis

All MRIs were acquired on the same 3T scanner. Sequences included T1-weighted (1 mm^3^ isotropic), T2-weighted (0.9 mm^3^ isotropic), FLAIR (1 mm^3^ isotropic) and proton density (PD) imaging (1.2 mm^3^ isotropic). Data acquisition and processing are described in the study protocol^9^ and online processing pipeline (DOI:10.7488/ds/7486).

All structural image sequences were coregistered to the baseline T2-weighted volume before segmentation.^13^ The intracranial volumes (ICV) were computationally generated from the baseline PD image. WMH volumes were acquired from FLAIR images using a semiautomatic approach. All segmentations were checked and manually edited if necessary. Old and acute infarcts were manually removed from WMH volumes. WMH volumes (mL) were expressed as %ICV to standardise for differences in brain and ICV. Total net WMH volume change was defined as 1 year minus baseline WMH volumes (%ICV).

## Statistical analysis

We used R V.4.2.2 (https://www.r-project.org) with packages *ggplot2* and *moments* to create figures and perform kurtosis and skewness calculations. We applied the four approaches (table 1) to the dataset and assessed how each method impacted the allocation of participants into change groups.

## Results

Baseline age was 65.5 years (SD=11.13), 67% were men, and mean baseline WMH volume was 15.5 mL (SD=19.19). WMH volume change over 1.05 years (SD=0.10) was available for 198 participants (attrition overview in online supplemental results). Mean net WMH volume change (mL), (online supplemental figure S1) was 0.98 mL (SD=2.84), range −7.98 to +12.84 mL. The distribution was leptokurtic (kurtosis=7.35) and positively skewed (skewness=1.51). The proportion of stable and regressing WMH, and range of WMH volume change varied substantially by definition (table 2). For example, % stable ranged from 16.7% to 55.6% and progression from 14.6% to 53.5%, while regression varied least from 28.3% to 29.8%.

**Table 2:** Participant distribution and WMH volume change (mL) after applying the four approaches to one dataset

Approach	N (%) regression	Regressing WMH change (mL)	N (%) stable	Stable WMH change (mL)	N (%) progression	Progressing WMH change (mL)
**Threshold 1**						
Mean volume (SD), (range)	59(29.8)	−1.36 (1.42)(−7.98 to –0.26)	110(55.6)	0.86 (0.89)(−0.23, 2.81)	29(14.6)	6.22 (3.07)(2.88, 12.84)
**Threshold 2**						
Mean volume (SD), (range)	59(29.8)	−1.36 (1.42)(−7.98 to –0.26)	33(16.7)	−0.02 (0.14)(−0.23, 0.22)	106(53.5)	2.59 (2.82)(0.25, 12.84)
**Percentile**						
Mean volume (SD), (range)	56(28.3)	−1.42 (1.43)(−7.98 to –0.33)	42(21.2)	−0.01 (0.18)(−0.28, 0.30)	100(50.5)	2.73 (2.85)(0.32, 12.84)
**Quintile**	**Q1**(**N=40**)	**Q2**(**N=40**)	**Q3**(**N=40**)	**Q4**(**N=39**)		**Q5**(**N=39**)
Mean (SD), (range)	−1.79 (1.54)(−7.98 to –0.68)	−0.27 (0.20)(−0.65, 0.03)	0.35 (0.18)(0.04, 0.70)	1.43 (0.48)(0.75, 2.33)		5.31 (3.07)(2.43, 12.84)

Q, quintile; WMH, white matter hyperintensity.

For the percentile approach, we calculated percentiles of WMH volume change in mL (described in online supplemental results, online supplemental table S1). The percentile closest to no change was percentile 39 (N=2, mean WMH volume change=0.001 mL (SD=0.004)).

We also applied the percentile and quintile approaches to WMH volume change as %ICV (approaches 1 and 2 cannot be applied since they use thresholds based on mL). Distribution of WMH volume (%ICV) and the results for percentile and quintile approaches are in online supplemental figure S2 and online supplemental tables S2,S3. The group sizes did not change compared with group sizes based on ‘raw’ mL, except that both approaches allocated two participants to different WMH change categories.

## Discussion

We compared four different approaches that define WMH volume change in one population and found that the proportion of participants per WMH category differs substantially between approaches.

The main difference was within the stable and progressing WMH categories. Based on approach 1, 14.6% of participants had WMH progression, compared with 53.5% (approach 2) and 50.5% (percentile approach), that is >3 times more progression according to approach 2 and the percentile approach, perhaps accounting for WMH progression dominance in the literature.^2^ The proportion of stable WMH is the largest in approach 1, influenced by the higher threshold required for progressing WMH. WMH regression occurred in around 30% of participants (approaches 1 and 2 and percentile approach).

Approach 1 was previously applied to three cohorts with cerebral small vessel disease (SVD) (follow-up times: 3, 2 and 5 years), with WMH progression occurring in 58.2%, 42.9% and 25.7% of the participants, respectively.^8^ Group size differences might reflect differences in data distribution and the use of >1 SD as threshold, which changes with mean WMH volume change. The reason for using this threshold is unclear.^12^ This might be based on ~68% of data falling within 1 SD from the mean for normally distributed data. When applied to WMH, this would suggest that 68% of the participants have stable WMH. However, as seen in online supplemental figures S1,S2 and other data,^4^ WMH volumes and volume change are rarely normally distributed, hence the >1 SD threshold is not recommended. A 0.25 mL threshold was also used previously to reflect the assumed minimum volume change visible on MRI, based on 1.5T MRI.^3 8 12^ However, this definition may not be suitable for MRI acquired at higher resolutions and field strengths, when smaller changes in volume may be detectable.

The effect that these thresholds can have is reflected in one study^8^ that applied a >1 SD decrease threshold to define WMH regression, in secondary analysis, resulting in no participants with WMH regression, suggesting that the SD threshold may be insensitive. The same study also assessed the effect of applying a >0.25 mL increase to define progression, which resulted in only one participant with stable WMH, indicating that fixed thresholds are too strict and not transferable between studies. Another influence might be time between scans. Approach 1 was applied to the same dataset (N=276) in different papers.^6 8^ While 2.2% of the participants had WMH regression over 5 years,^8^ 11.2% had WMH regression after 8.7 years.^6^

Advantages of the quintile and percentile approaches are that they can be applied to any data distribution and to both ‘raw’ WMH volumes (mL) and corrected WMH volumes (e.g., %ICV). A disadvantage is that the volume ranges within quintiles and percentiles from one dataset may not overlap with those of another dataset as no set thresholds are used and assess relative differences. However, they permit the comparison of clinical outcomes between those with the most progression and most regression by providing a distribution of smallest to largest net change in WMH. The quintile approach does not define specific progression, stable and progression categories since these will differ per population. Rather it provides granularity to test associations with WMH volume change that is clinically practical. Measurement uncertainties, that is, test–retest repeatability, might play a role in comparing studies and might limit defining change. These uncertainties can differ between study and MRI protocol and depend on several factors, including acquisition and analysis methods.

Study strengths include applying four WMH volume change definitions to one prospective population for the first time, use of well-validated methods throughout and an in-depth consideration of the definitions and their implications. Limitations include using one study population with minor stroke; other populations should be evaluated to determine the optimal WMH change definition in a range of populations reflecting common clinical presentations of SVD, for example, from memory clinics and people with a more severe stroke.

Future research should assess the relation of thresholds to clinical presentation, follow-up times, risk factors, clinical outcomes as WMH volume change might differ per population.^2^ So far, the approaches were applied to neurology outpatients with SVD neuroimaging features, lacunar stroke and sporadic SVD^6 8 12^ (minor), ischaemic stroke patients,^3 5 7^ people with normal cognition or mild cognitive impairment^4 14^ and community-dwelling older people.^15^

To summarise, WMH change definitions have a major effect on the proportion of a sample that will be classed as regressing, stable or progressing WMH. We encourage using the percentile and quintile approaches as they accommodate non-normal data distributions, reflect relative differences across a population and can use raw mL or %ICV. More studies should compare WMH change definitions to work towards a consensus on clinically relevant definitions to ensure consistent study outcomes.

## Supplementary material

10.1136/svn-2024-003300 online supplemental file 1
