# Supplementary material for: Definitions of white matter hyperintensity change: impact on estimates of progression and regression
Source: Stroke Vasc Neurol. 2024 Oct 2;10(3):e003300. doi: 10.1136/svn-2024-003300 (PMC12230215; doi:10.1136/svn-2024-003300)
Supplement: online supplemental file 1 [file svn-10-3-s001.pdf]

Supplemental material

Supplemental Figures

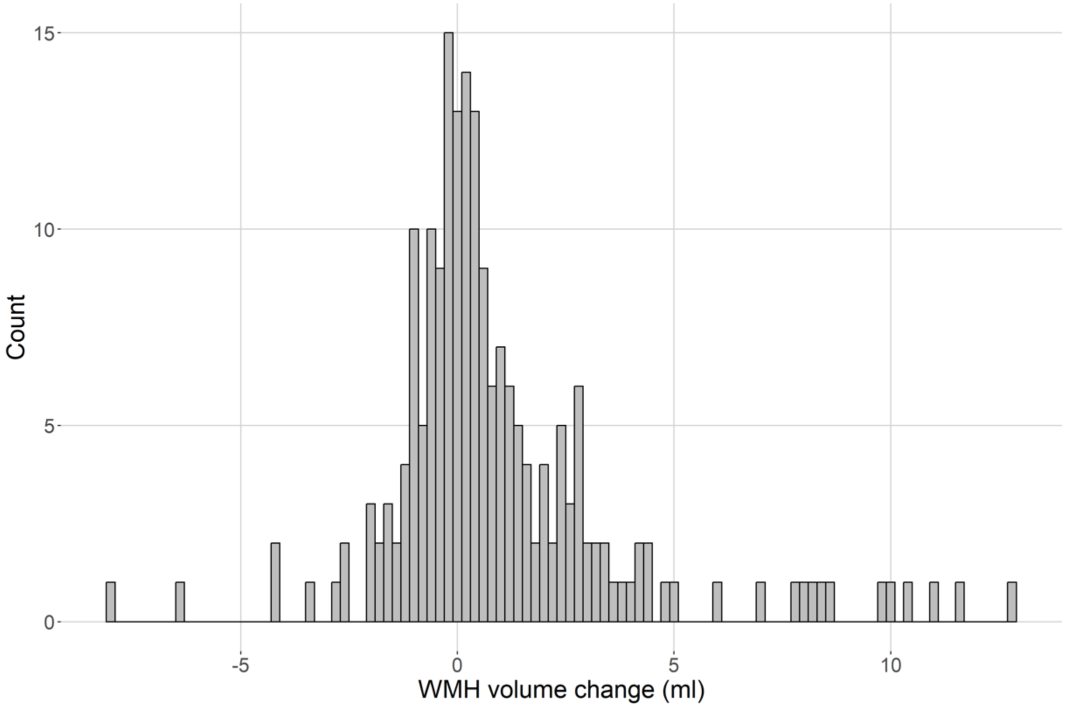

Figure S1. Distribution of WMH volume change (ml) over one year. WMH: white matter hyperintensity

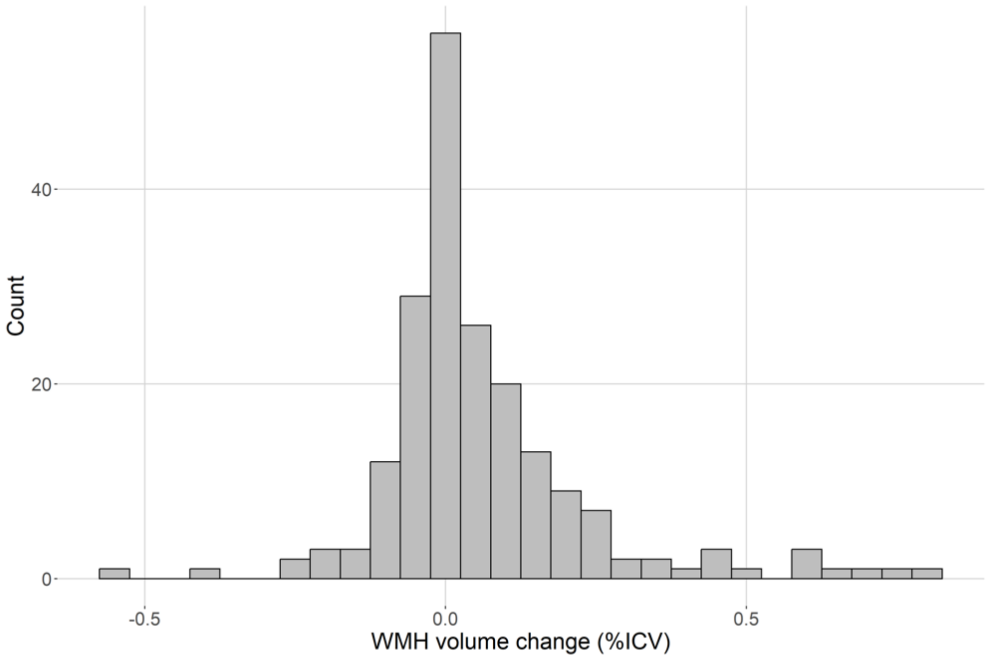

Figure S2. Distribution of WMH volume change (%ICV). ICV: Intracranial volume; WMH: White matter hyperintensity

Supplemental Results

Overview missing WMH volumes at one-year visit

N=230 participants were recruited into the study. After the one-year visit, WMH change volume were available for n=198. Reasons for missing scans (n=32) were:

- Deceased n=2
- Illness and unable to participate in study n=5
- Declined MRI scan n=8
- Phone follow-up only (due to COVID-19) n=6
- Declined follow-up altogether n=1
- Not contactable despite multiple attempts n=6
- Poor imaging quality and not analysable n=4

Overview of percentile approach

The WMH volume change was divided into percentiles and the percentile with least or no WMH volume change was identified. That percentile and the 10 percentiles below and above that percentile of 'no change' were combined and defined as the group with 'stable WMH' (Table S1). All percentiles above the +10 percentiles of no change were included in the progression group and all percentiles below -10 percentile of no change were included in the regression group. This resulted in a definition of stable WMH with a range of -0.15 to 0.15 ml (percentiles 25 – 45). WMH progression is defined as an increase of more than 0.15 ml (percentile 46-100) and WMH regression a decrease of more than 0.15 ml (percentile 0-24).

Supplemental Tables

Table S1. WMH volume change

	Change in ml			Change in %ICV		
Percentile	N	Mean	SD	N	Mean	SD
1	2	-7.15	1.18	2	-0.479	0.119
2	2	-4.22	0.082	2	-0.244	0.021
3	2	-3.06	0.334	2	-0.205	0.027
4	2	-2.6	0.032	2	-0.164	0.021
5	2	-2.04	0.013	2	-0.138	0.004
6	2	-1.94	0.102	2	-0.116	0.002
7	2	-1.67	0.069	2	-0.108	0.007
8	2	-1.54	0.043	2	-0.101	0.000
9	2	-1.4	0.137	2	-0.093	0.000
10	2	-1.28	0	2	-0.086	0.001
11	2	-1.19	0.015	2	-0.08	0.000
12	2	-1.06	0.024	2	-0.07	0.004
13	2	-1.01	0.034	2	-0.064	0.004
14	2	-0.952	0.016	2	-0.06	0.000
15	2	-0.935	0.003	2	-0.057	0.003
16	2	-0.916	0.02	2	-0.054	0.001
17	2	-0.796	0.049	2	-0.052	0.000
18	2	-0.746	0	2	-0.048	0.003
19	2	-0.703	0.017	2	-0.043	0.001
20	2	-0.683	0.003	2	-0.041	0.001
21	2	-0.626	0.03	2	-0.038	0.004
22	2	-0.578	0.034	2	-0.034	0.001
23	2	-0.54	0.002	2	-0.033	0.001
24	2	-0.505	0.017	2	-0.031	0.001
25	2	-0.464	0.005	2	-0.027	0.001
26	2	-0.425	0.012	2	-0.025	0.000
27	2	-0.4	0.02	2	-0.024	0.001
28	2	-0.355	0.04	2	-0.022	0.001
29	2	-0.278	0.004	2	-0.017	0.003
30	2	-0.246	0.017	2	-0.014	0.001
31	2	-0.209	0.02	2	-0.013	0.000
32	2	-0.171	0.001	2	-0.01	0.000
33	2	-0.163	0.006	2	-0.009	0.000
34	2	-0.141	0.021	2	-0.009	0.000
35	2	-0.12	0	2	-0.008	0.000
36	2	-0.1	0.003	2	-0.006	0.001
37	2	-0.072	0.026	2	-0.004	0.002
38	2	-0.028	0.016	2	-0.002	0.001
**39**	**2**	**0.001**	**0.004**	**2**	**0**	**0.000**
40	2	0.026	0.004	2	0.001	0.000
41	2	0.043	0.011	2	0.003	0.001
42	2	0.075	0.002	2	0.005	0.000
43	2	0.129	0.016	2	0.008	0.001
44	2	0.161	0.017	2	0.011	0.001
45	2	0.201	0.012	2	0.012	0.000
46	2	0.219	0.007	2	0.015	0.000
47	2	0.261	0.011	2	0.016	0.000
48	2	0.276	0.004	2	0.017	0.002

49	2	0.293	0.007	2	0.019	0.000
50	2	0.327	0.006	2	0.02	0.000
51	2	0.349	0.004	2	0.021	0.000
52	2	0.366	0.009	2	0.022	0.000
53	2	0.389	0.007	2	0.023	0.000
54	2	0.412	0.021	2	0.026	0.002
55	2	0.475	0.016	2	0.028	0.001
56	2	0.502	0.009	2	0.031	0.000
57	2	0.536	0.022	2	0.033	0.002
58	2	0.586	0.022	2	0.037	0.002
59	2	0.64	0.021	2	0.041	0.002
60	2	0.699	0	2	0.044	0.000
61	2	0.779	0.042	2	0.048	0.003
62	2	0.851	0.019	2	0.053	0.000
63	2	0.88	0.002	2	0.055	0.002
64	2	0.926	0.008	2	0.06	0.003
65	2	0.999	0.061	2	0.064	0.001
66	2	1.08	0.022	2	0.067	0.000
67	2	1.11	0.022	2	0.072	0.006
68	2	1.16	0.033	2	0.078	0.001
69	2	1.24	0.002	2	0.08	0.000
70	2	1.33	0.04	2	0.081	0.001
71	2	1.44	0.018	2	0.088	0.000
72	2	1.48	0.016	2	0.092	0.001
73	2	1.52	0.02	2	0.096	0.003
74	2	1.68	0.026	2	0.111	0.001
75	2	1.86	0.004	2	0.115	0.002
76	2	1.94	0.023	2	0.121	0.002
77	2	2.01	0.027	2	0.125	0.002
78	2	2.17	0.002	2	0.13	0.001
79	2	2.31	0.005	2	0.141	0.001
80	2	2.38	0.071	2	0.147	0.005
81	2	2.5	0.023	2	0.153	0.002
82	2	2.64	0.031	2	0.162	0.003
83	2	2.74	0.018	2	0.17	0.000
84	2	2.79	0.015	2	0.18	0.003
85	2	2.85	0.047	2	0.183	0.001
86	2	2.98	0.016	2	0.192	0.002
87	2	3.22	0.064	2	0.209	0.000
88	2	3.42	0.023	2	0.228	0.009
89	2	3.66	0.059	2	0.237	0.003
90	2	4.11	0.115	2	0.248	0.004
91	2	4.37	0.106	2	0.266	0.009
92	2	4.66	0.254	2	0.286	0.007
93	2	5.5	0.739	2	0.355	0.019
94	2	7.41	0.636	2	0.415	0.045
95	2	8.07	0.156	2	0.467	0.003
96	2	8.43	0.098	2	0.537	0.056
97	2	9.85	0.096	2	0.588	0.013
98	2	10.8	0.428	2	0.673	0.057
99	1	11.7	-	1	0.753	-
100	1	12.8	-	1	0.809	-

Darkest row marks percentile of no change. Rows in grey reflect the -/+ 10 percentiles included in the 'stable WMH' group.

Table S2. WMH volume change (%ICV) categorised using the percentile approach

	N	WMH regression	N	Stable WMH	N	WMH progression
Percentiles %ICV, mean (SD), [range]	56	-1.42 (1.43) [-7.98, -0.33]	42	0.01 (0.18) [-0.28, 0.33]	100	2.73 (2.85) [0.28, 12.84]

ICV: Intracranial volume; WMH: White matter hyperintensity

Table S3. WMH volume change (%ICV) categorised using the quintile approach

	Q1 (N=40)	Q2 (N=40)	Q3 (N=40)	Q4 (N=39)	Q5 (N=39)
Quintiles mean (SD), [range]	-1.79 (1.54) [-7.98, -0.68]	-0.27 (0.20) [-0.65, 0.03]	0.35 (0.18) [0.04, 0.70]	1.50 (0.64) [0.70, 2.49]	5.33 (3.11) [2.30, 12.84]

ICV: Intracranial volume; WMH: White matter hyperintensity
